# PRELIMINARY FEASIBILITY OF AN MHEALTH INTERVENTION FOR OLDER ADULTS WITH SERIOUS MEDICAL ILLNESS AND CAREGIVERS

**DOI:** 10.1093/geroni/igae098.2905

**Published:** 2024-12-31

**Authors:** Danielle Llaneza, Elissa Kozlov

**Affiliations:** University of Houston, Houston, Texas, United States; Rutgers, The State University of New Jersey, Piscataway, New Jersey, United States

## Abstract

Managing serious illness is often distressing and time-consuming for older adults and their caregivers. Up to 70% of adults with serious illness have symptoms of anxiety. Additionally, patients with high symptom burden and anxiety heavily impact family caregivers, which nearly 8 million older adults in the U.S. rely on for assistance. Mindfulness Therapy (MT) is a promising, non-pharmacological technique with proven efficacy and effectiveness in managing stress and anxiety in diverse populations. Mindfulness Coach is an mhealth delivered mindfulness therapy intervention developed by the Veterans Affairs National Center. The objective of this study is to report the preliminary feasibility of an eight-week trial of mHealth-delivered mindfulness therapy to alleviate anxiety and stress symptoms in this population. Thirty-one older adults with serious medical illness or their caregivers were recruited to participate in this study. After receiving an orientation to using the app, participants were instructed to complete two levels per week to learn about and practice mindfulness skills. At the end of the eight weeks, the Mindfulness Coach participant users ranged from zero minutes to 1154 minutes with an average use of 173.4 minutes. Additionally, changes in anxiety levels were also observed. This study offers preliminary evidence that an mHealth mindfulness app is being utilized by older adults with a serious medical illness or their caregivers to learn mindfulness skills to help manage the stress and anxiety associated with living with a serious medical condition.

